# Affibody-DyLight Conjugates for *In Vivo* Assessment of HER2 Expression by Near-Infrared Optical Imaging

**DOI:** 10.1371/journal.pone.0041016

**Published:** 2012-07-20

**Authors:** Rafal Zielinski, Moinuddin Hassan, Ilya Lyakhov, Danielle Needle, Victor Chernomordik, Alejandra Garcia-Glaessner, Yasaman Ardeshirpour, Jacek Capala, Amir Gandjbakhche

**Affiliations:** 1 Radiation Oncology Branch, Center for Cancer Research, National Cancer Institute, National Institutes of Health, Bethesda, Maryland, United States of America; 2 Department of Molecular Biology, The John Paul II Catholic University of Lublin, Lublin, Poland; 3 Section on Analytical and Functional Biophotonics, Program on Pediatric Imaging and Tissue Sciences, Eunice Kennedy Shriver National Institute of Child Health and Human Development, National Institutes of Health, Bethesda, Maryland, United States of America; 4 Protein Chemistry Laboratory, SAIC-Frederick, Inc., National Cancer Institute, Frederick, Maryland, United States of America; 5 VARNISS, L.L.C., Frederick, Maryland, United States of America; 6 Center for Cancer Research, Nanobiology Program, National Cancer Institute, Frederick, Maryland, United States of America; 7 Protein Engineering Section, Macromolecular Crystallography Laboratory, NCI, Frederick, Maryland, United States of America; City of Hope National Medical Center and Beckman Research Institute, United States of America

## Abstract

**Purpose:**

Amplification of the HER2/neu gene and/or overexpression of the corresponding protein have been identified in approximately 20% of invasive breast carcinomas. Assessment of HER2 expression *in vivo* would advance development of new HER2-targeted therapeutic agents and, potentially, facilitate choice of the proper treatment strategy offered to the individual patient. We present novel HER2-specific probes for *in vivo* evaluation of the receptor status by near-infrared (NIR) optical imaging.

**Experimental Design:**

Affibody molecules were expressed, purified, and labeled with NIR-fluorescent dyes. The binding affinity and specificity of the obtained probe were tested *in vitro*. For *in vivo* validation, the relationship of the measured NIR signal and HER2 expression was characterized in four breast cancer xenograft models, expressing different levels of HER2. Accumulation of Affibody molecules in tumor tissue was further confirmed by *ex vivo* analysis.

**Results:**

Affibody-DyLight conjugates showed high affinity to HER2 (K_D_ = 3.66±0.26). No acute toxicity resulted from injection of the probes (up to 0.5 mg/kg) into mice. Pharmacokinetic studies revealed a relatively short (37.53±2.8 min) half-life of the tracer in blood. Fluorescence accumulation in HER2-positive BT-474 xenografts was evident as soon as a few minutes post injection and reached its maximum at 90 minutes. On the other hand, no signal retention was observed in HER2-negative MDA-MB-468 xenografts. Immunostaining of extracted tumor tissue confirmed penetration of the tracer into tumor tissue.

**Conclusions:**

The results of our studies suggest that Affibody-DyLight-750 conjugate is a powerful tool to monitor HER2 status in a preclinical setting. Following clinical validation, it might provide complementary means for assessment of HER2 expression in breast cancer patients (assuming availability of proper NIR scanners) and/or be used to facilitate detection of HER2-positive metastatic lesions during NIR-assisted surgery.

## Introduction

Amplification of the HER2/neu gene and/or overexpression of the corresponding protein have been identified in invasive breast, non-small cell lung, and ovarian carcinomas as well as B-cell acute lymphoblastic leukemia [1], [2]. Particularly in breast cancer, elevated HER2 is associated with increased proliferation and survival of cancer cells and, thereby, contributes to poor therapy outcomes and unfavorable prognoses [3], [4]. Therefore, accurate evaluation of HER2 status in breast cancer patients is a key factor in determining their further treatment. Women with HER2-positive tumors qualify for antibody-based targeted therapy (trastuzumab) alone, or in combination with chemotherapy [5]. Clinical evaluation of HER2 expression is based on IHC or FISH staining of biopsied tissue. Both methodologies are *ex vivo* techniques and, due to tumor heterogeneity, often deliver false-positive or -negative results [6].

Affibody molecules constitute a unique class of artificial ligands. They are relatively small (∼7 kDa) affinity proteins, structurally based on a 58-amino-acid scaffold derived from the Z domain of the *Staphylococcus aureus* protein A using combinatorial protein engineering [7], [8]. HER2-specific Affibody molecules strongly bind extra cellular domain (ECL) of human HER2 (K_D_ = 22 pM), without affecting the receptor activation status [9]. Importantly, HER2-Affibody molecules bind to a domain distinct from the domain that trastuzumab or pertuzumab bind [10].

It has been shown that Affibody molecules, labeled with radionuclides such as ^99m^Tc, ^111^In, ^68^Ga, ^90^Y, ^125^I, and ^18^F, could be successfully applied to SPECT and PET imaging [11]–[17]. Recently, we and other groups have reported that HER2- and EGFR-specific Affibody molecules, fused to fluorescent proteins or labeled with reporter enzymes, were successfully applied to assess receptor expression in cell culture and *ex vivo* samples [18], [19]. HER2-specific Affibody molecules (Z_HER2_) have also been used as targeting vectors in HER2-targeted thermosensitive liposomes for local, hyperthermia-triggered release of the content in the tumor [20], [21]. The same molecules were incorporated, as a targeting module, into HER2-Affitoxin, a recombinant protein, designed to deliver *Pseudomonas exotoxin* A to HER2-overexpressing cells [22] and tumors [23].

Optical imaging is a powerful tool allowing *in vivo* analysis of macroscopic distribution of fluorescent labels [24]–[26]. The serious limitation of that methodology in the optical spectral range is high tissue autofluorescence and limited penetration of the tissue by the visible light. However, introduction of Near-Infrared fluorescent beacons, similar to DyLight-750, significantly eased these limitations. Over the past several years, there has been an explosion of reports describing successful *in vivo* NIR fluorescence imaging using antibodies, antibody fragments, or small molecules as contrast agents.

In our previous work, we have observed a considerable accumulation of a HER2-specific probe, consisting of (Z_HER2∶342_)_2_ Affibody molecule, albumin binding domain (ABD), and AlexaFluor-750 (ABD-(Z_HER2∶342_)_2_-AlexaFluor750), in subcutaneous BT-474 xenografts [27]. Our subsequent studies, have shown that a sequence of fluorescence images, obtained with this tracer, can be used to monitor *in vivo* changes of receptor expression following treatment with an HSP90 inhibitor, 17-Dimethylaminoethylamino-17-demethoxygeldanamycin (17-DMAG) [28]. However, due to the relatively long half-life of ABD-(Z_HER2∶342_)_2_-AlexaFluor750 in the blood (∼40.5 hours), quantification of HER2 expression requires a long observation time, which limits its utility in preclinical and, especially, potential clinical studies.

The goal of the current work was to create and characterize an Affibody-based fluorescent probe that would allow assessment of HER2 expression *in vivo*, during a much shorter observation time. The new probe, Z_HER2_-DyLight750, combining a monovalent Affibody molecule with DyLight-750, binds specifically to HER2 with high affinity both *in vitro* and *in vivo.* As expected, its relatively fast pharmacokinetics allowed us to reduce the observation time necessary for *in vivo* characterization of HER2 expression to 3.5 hours, which constitutes a significant improvement as compared to ∼15 hours in the case of (ABD-(Z_HER2∶342_)_2_-AlexaFluor750) [27].

## Materials and Methods

### HER2- and Taq-specific Affibody Cloning, Overexpression, and Purification

Z_HER2_ Affibody molecule containing a flexible spacer Gly4-Ser-Gly2 followed by a unique cysteine amino acid residue at the C-terminus, and a hexahistidine tag at the N-terminus (Z_HER2_-GS-Cys), was expressed as an N-terminal fusion with Maltose Binding Protein (MBP) separated from the hexahistidine tag by the Tobacco Etch Virus (TEV) [29] proteinase cleavage site ENLYFQG (MBP-TEV-His6-Z_HER2_-GS-Cys), using expression vector pMTZGC1 [21]. The similar protein containing Z_Taq_ Affibody molecule (Z_Taq_-GS-Cys) instead of Z_HER2_ was produced using the expression vector pTC1.

Both, HER2- and Taq-Affibody molecules fused to Maltose Binding Protein, were expressed in One Shot® BL21 Star™ (DE3) cells (Invitrogen Corporation, Carlsbad, CA). Transformed bacterial cells were cultured in a Super Broth (Quality Biological, Gaithersburg, MD) medium, supplemented with ampicillin at 100 µg/ml (Sigma-Aldrich, St. Louis, MO). Protein expression was induced when the OD_600_ value reached 1 AU by addition of Isopropyl-β-D-Thiogalactopyranoside to the final 1**mM concentration (IPTG, MP Biomedicals, Solon, OH). Bacteria were centrifuged (4,000×G, 10 min) three hours post induction, resuspended and subjected to two sonication cycles, 5 min each, separated by a 5-min cooling step. The lysis step was performed in an ice-cold PBS buffer supplemented with a 1×proteinase inhibitors cocktail (Complete Stop, Roche Indianapolis, IN) using Sonic Dismembrator (Fisher Scientific, Pittsburgh, PA). Cell debris were removed by centrifugation (48,000×G, 20 min); supernatant was filtered through 0.22 µm filters and loaded onto a 5 ml HisTrap column. Next, unbound protein was washed out following elution with a linear gradient of imidazole from 0 to 300**mM (Sigma-Aldrich, St. Louis, MO) in phosphate buffer (pH 8). Eluted fractions were combined based on an electrophoretic mobility, followed by a buffer exchange to PBS containing 1 mM Dithiothreitol (DTT) (Sigma-Aldrich, St. Louis, MO) and overnight digestion with recombinant TEV cysteine protease at room temperature as described in Kapust et al. [29]. Affibody molecules containing a hexahistidine tag were re-purified on a 1 ml HisTrap column as described above. Protein fractions containing Affibody were combined, imidazole-containing buffer was exchanged to PBS followed by 0.22**µm filtration.

### Modification of Z_HER2_- and Z_Taq_ Molecules

For NIR optical imaging, Z_HER2_- and Z_Taq_ Affibodies were labeled with a maleimide derivative of DyLight-750 or DyLight-488 (Pierce, Rockford, IL) by attaching the dye to the C-terminal cysteine of Affibody molecules. Before the conjugation step, both Affibody molecules were reduced by incubation with tris(2-carboxyethyl)phosphine (TCEP) for 30 min at 4°C and, then, mixed with a 4-fold excess of DyLight-Maleimide derivatives. The reaction was carried out at 4°C overnight. Labeled Affibodies were re-purified on a HisTrap column to remove unreacted dye. After purification, the imizadole-containing buffer was changed to PBS, followed by 0.22-µm filtration. Labeling efficacy was tested spectrophotometrically according to the manufacturer’s protocol.

### Cell Culture

The human breast cancer cell lines: BT-474, SK-BR-3, MDA-MB-361, MDA-MB-468, MCF-7 and human lung fibroblast WI-38, were obtained from the American Type Culture Collection (ATCC, Manassas, VA). The cells were grown in RPMI (BT-474, WI-38), DMEM-F12 (MDA-MB-468, SK-BR-3) or DMEM (MDA-MB-361, MCF-7) culture media supplemented with 10% fetal bovine serum (FBS) and 1% Pen/Strep (10,000 U penicillin, 10 mg/ml streptomycin) at 37°C at 5% CO_2_ in a humidified environment. A solution of 0.05% trypsin and 0.02% EDTA (Invitrogen, Carlsbad, CA) in PBS was used for cells detachment.

### Flow Cytometry

BT-474 cells were trypsinized, counted and aliquoted to 3×10^5^ cells per analyzed point. For estimation of the binding, affinity the cells were incubated with an increasing concentration of DyLight-488-labeled Z_HER2_-GS-C or Z_Taq_-GS-Cys in PBS containing 2% FBS. In competition assay, the cells were pre-titrated with competitors: Z_HER2_-GS-Cys and Z_HER2_-Cys followed by incubation with fixed concentration (51 nM) of DyLight-488-labeled Z_HER2_-Cys. The reaction was carried out on ice with intensive shaking for one hour. Next, the cells were washed three times with PBS and analyzed by an LSR II flow cytometer using blue laser (λ_emission_  = 488 nm) with 505 nm long pass and 525/50 nm band pass filters set. FACSDiVa software (BD Biosciences, San Jose, CA), was used for data acquisition and FlowJo (Tree Star Inc., Ashland, OR) for data analysis. Three independent experiments were performed. K_D_ for Z_HER2_-GS-Cys labeled with DyLight-488 was calculated using GraphPad Prism (GraphPad Software, Inc., San Diego, CA) after subtraction of non-specific binding. In competition assay, each point was normalized to median fluorescence intensity (MFI) of cells exposed to the probe without any competitor. IC_50_ values for Z_HER2_-GS-Cys and Z_HER2_-Cys were obtained by GraphPad Prism using sigmoid equation with Hill slope.

### Confocal Microscopy

HER2-positive and HER2-negative cells (SK-BR-3 and MDA-MB-468, respectively) were plated on an 8-chamber cover slip (Thermo Scientific, Rochester, NY) at the density of 2×10^4^ cells/chamber. After overnight attachment, Affibody-DyLight-488 conjugate was added to the cells at 0.5 µg/ml. After 30 min of incubation, the cells were washed and further incubated in regular growth medium for the indicated time in 37°C. One hour before imaging, the cells were exposed to Hoechst 33342 at 2 µg/ml (Invitrogen, Carlsbad, CA) for nuclei counterstaining. Then, the cells were rinsed three times with PBS and imaged using Zeiss LSM 510 confocal microscope (Carl Zeiss Inc., Thornwood, NY) with an Axiovert 100 M inverted microscope, using an 80 mW argon UV laser tuned to 364 nm and a 25 mW argon laser tuned to 488 nm. A 40×1.3NA Plan-Apochromat oil immersion objective and a multi-track configuration were applied. DyLight-488 and Hoechst signals were collected with a BP 505–550 filter and a BP 385–470 filter after excitation with 488 nm and 364 nm laser lines, respectively. Images (8 bit, 512×512 pixels) were acquired with a scan zoom of 2.5 and a line average of 4 using the Zeiss AIM software.

### Western Blot

Starved SKBR3 cells were exposed for 30 min to Affibody molecules and their fluorescent derivative at 10 µg/ml. For positive control cells were treated with EGF at 100 ng/ml. 20 µg of cell lysates was separated in 4–12% NU PAGE followed by transfer on PVDF membrane. Immunodetection was performed using anti-phosphorylated MAPK (D13.14.4E) and AKT (244F9) antibodies. Anti-total MAPK (137F5) and anti-total AKT (11E7) were used for detection of the protein is cell lysates. All Antibodies were from Cell Signaling Technology (Danvers, MA).

### Animal Models

Female athymic nude mice (*nu/nu* genotype, BALB/c background), 5 to 8 weeks old, were purchased from the Animal Production Program (NCI, Frederick, MD). This study was approved by the Animal Safety and Use Committee of NIH (Animal Study Proposal: ROB 117). Mice were cared for and treated in accordance with the Department of Health and Human Services Guide for the Care and Use of Laboratory Animals.

The tumors were initiated by subcutaneous (s.c.) injection of 5–8 million cells, suspended in 0.1 ml of 30% Matrigel solution (BD Biosciences, Bedford, MA), into the right forelimb. Growth of BT-474, MDA-MB-361, and MCF-7 cell lines was facilitated by s.c. implantation of estrogen pellets (0.72 mg, 90 days release, Innovative Research of America, Sarasota, FL) 24 hours prior to cells injection. Imaging studies were initiated when tumor diameter reached 0.5–0.8 cm.

### Immunohistochemistry Analysis

Immunohistochemical service was provided by the Pathology/Histotechnology Laboratory, SAIC-Frederick, Inc. Tissue specimens from xenografts were fixed in 10% neutral-buffered formalin. Five-micrometer, paraffin-embedded sections were immobilized on positively charged slides and Affibody staining was conducted on Leica Microsystems Bond Autostainer (Leica), followed by a citrate buffer antigen-retrieval step. After a blocking step in 2% normal rabbit serum (Vector Laboratories Burlingame, CA), slides were exposed to goat anti-Affibody antibody (Abcam, Cambrige, MA) for 30 minutes at a dilution of 1∶100. Signal detection was conducted using a Bond Intense R Detection Kit after 1-hour incubation with biotinylated rabbit anti-goat IgG (Vector Laboratories, Burlingame, CA), at a dilution of 1∶100. HER2 detection was conducted after immobilized slides were deparaffinized and rehydrated using a DAKO HercepTestTM (Dako, Carpinteria, CA). Both Affibody- and HER2-stained slides were counterstained according to Gill’s hematoxylin staining protocol. Slides were visualized by Aperio Scanscope XT (Aperio, Vista, Ca) with 20× objective. Digital images are annotated via the companion Aperio Imagescope software (software version 11.1.2).

### ELISA–Enzyme-Linked ImmunoSorbent Assay

To determine the HER2 protein level in the tumor tissue, the ELISA test was performed. Animals were sacrificed at the end of optical imaging, followed by tumor tissue extraction. One part of the tumor was flash-frozen in liquid nitrogen and stored at -80°C. The HER2 level was measured using the c-ErbB2/c-Neu Rapid Format ELISA Kit (EMD Chemicals, Gibbstown, NJ), following the protocol provided by the manufacturer. Protein concentration in the tissue lysates was measured using BCA Protein Assay Kit (Pierce, Rockford, IL) according to manufacturer protocol. Data are expressed as nanogram of HER2 per milligram of tissue lysate ± SEM.

### Near-Infra-Red Optical Imaging

Near-Infra-Red optical imaging was performed using a previously described NIR fluorescence small-animal imager [30]. The system is based on a time-domain technique, where an advanced time-correlated, single-photon counting device is used in conjunction with a high-speed repetition-rate tunable laser to detect individual photons. It contains a photomultiplier tube used as a detector, a temperature-controlled scanning stage with an electrocardiogram, and temperature monitoring device for small animals. The scanning head consists of multimode optical fibers that are used to deliver light from an excitation source and an emitted fluorescence signal to detectors. The imager has a laser source for fluorescence excitation (λ = 750 nm), an emission filter (λ = 780 nm) for fluorescence detection, and a computer for data analysis. The imager scans in a raster pattern over the skin or other tissue surfaces to produce a real-time two-dimensional image of the region of interest (ROI). A cooled, charge-coupled device (CCD) camera is used to guide the scan to the ROI and to measure the fluorescence intensity distribution.

For fluorescence imaging, mice were anesthetized by inhalation of isoflurane (5% for induction, 2–2.5 for anesthesia maintenance, oxygen flow 1 l/min). Then, 10 µg of Affibody-DyLight-750 conjugate in 100 µl of PBS were injected intravenously, and the ROI was imaged at several predetermined time points.

To assess the tumor-specific fluorescence, the mean and standard deviation of the fluorescence signal were obtained by averaging of the maximum pixel values (16 pixels) over the ROIs at the center of the tumor and in normal tissue at the corresponding contralateral site. In the series of experiments, aimed at quantification of the time courses of fluorescence the measurements started 10 min after injection and were repeated at 10 time points, approximately at *t_i_*  = 0.17 0.5, 1, 1.5, 2, 2.5, 3, 4, 6 and 24 hours. The tumor- specific fluorescence data were obtained from BT-474, MDA-MB-361, and MCF-7 xenografts, expressing different levels of HER2.

The half-life of the probe in the circulation (the time of the probe concentration decrease to a half of its maximum value), was estimated using average fluorescence intensities from the contra-lateral site of the animals at given time points. The experimental data were fitted to a single-exponent decay function, 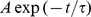. The washout time of the probe is related to the probe half-life by the following equation: 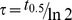.

In addition to a highly accurate in-house scanning system, providing quantitative data from the relatively small ROI, we have used a commercial NIR-optical imager, Pearl Impulse Imaging System (LI-COR Biosciences), to qualitatively investigate Z_HER2_ distribution at early time points post injection in the whole mouse. This instrument is equipped with two lasers, 685 and 785 nm, for excitation and two emission filters, λ_em_ = 720 and 820 nm, respectively. A thermoelectrically-cooled CCD camera was used for signal detection. Before the imaging, 0.28 mm polyethylene catheter (BD Franklin Lakes, NJ) with 30 G needle was placed in lateral tail vein of mice with subcutaneous BT-474 xenografts. The animals were positioned laterally in the imager and 10 µg of Affibody-DyLight-750 conjugate was administered immediately after beginning of acquisition. Series images were taken every second for one minute. Two frames per minute were acquired over the following two hours. For longer time points, the mice were imaged at 24, 48, 72 hours and 5 days post probe administration. During imaging, the mice were kept anesthetized with a 1% isoflurane/oxygen mixture, and sacrificed at the end of experiment.

### Statistical Analysis

Estimates of the binding affinity parameters are based on three independent experiments and are presented as an average K_D_ or IC_50_ value ± SD. For evaluation of the probe accumulation in the xenograft (mouse model) total number of 25 mice has been used (n = 3–6 per tumor model). Half-life of HER2-specific and non-specific probes is presented as an average ± SEM (Standard Error of the Mean). Additionally, 95% confidence interval was calculated for each individual animal. Probe accumulation in the tumor was evaluated separately for each time point as outlined above, and is expressed as a mean value ± SEM.

### Evaluation of HER2 Expression in vivo Using our Mathematical Model

According to our compartmental kinetic model, previously described in [28], [31], the quantitative information about HER2 expression level can be extracted from the time dependence of fluorescent intensity evaluated from the time series of images. More specifically, the initial growth rate of the fluorescence intensity is proportional to the starting rate of the probe accumulation in the tumor area after the probe injection. In contrast to our previous work we used here considerably shorter time scale of the temporal fluorescence variations after the probe injection, due to smaller size of the probe and the resulting significantly faster washout times.

The measurements of the fluorescence in the normal tissue on contralateral side have shown that after 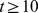 min, the probe concentration in blood circulation decreases with time exponentially 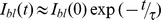, with the washout time, 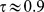
h. Additional fluorescence intensity, 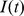, coming from the tumor area, originates from the bound fluorescent ligands and free ligands either in the tumor tissue or extra blood circulation due to angiogenesis. Intensity 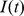, can be estimated as a difference between the fluorescent signal from the tumor and that of the contralateral side. In its turn, the latter rate depends linearly on the concentration of the binding sites for the specific probe, i.e., HER2 expression. To take into account uncertainties in the concentrations of the free ligands in the tumor area of individual mice, we have introduced a normalized rate of accumulation (NRA) [31]. The blood contribution of the tumor’s extra vasculature has been subtracted from the measured signal, assuming that at early times 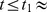 0.5 h after the injection most of the probe is in the blood, and corresponding signal decreases exponentially with characteristic time 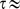
0.9 h.

We limit our analysis here to the initial phase of the probe accumulation 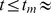
3.5 h, when the ligand-receptor dissociation can likely be disregarded (the dissociation rate constant k_off_ is assumed to be sufficiently low). In this case, for the first approximation, the theoretical model, describing probe accumulation at the tumor area, can be presented by the generic equation:
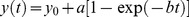
(1)Where, in accordance with the kinetic model [31] variable 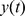 corresponds to the concentration of bound ligands in the tumor,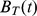, parameter *a* is proportional to the total concentration of HER2 receptors in the tumor, 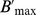, i.e., HER2 expression, while parameter *b* is proportional to the binding rate of fluorescent ligands to the receptors and the concentration of free ligands in the tumor tissue 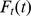. Thus, initial rate of accumulation of HER2-specific fluorescent ligands at early times 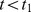 (i.e,., the product *ab* from Eq. (1)), is proportional to 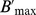
and concentration of free ligands in tissue, 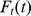, i.e., 
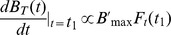
, or, assuming kinetic equilibrium between free ligands in blood and tumor tissue, 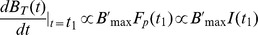.To reduce uncertainties related to slightly different concentrations of the ligands in the blood circulation of individual mice due to variations in the probe doses and blood volume, all fluorescence intensities were normalized to the measured value of intensity at the contralateral side at 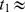
0.5 h. Fitting of the normalized temporal data to the model Eq. (1) allows to estimate NRA, given by the product *ab*.

## Results

**Figure 1:**
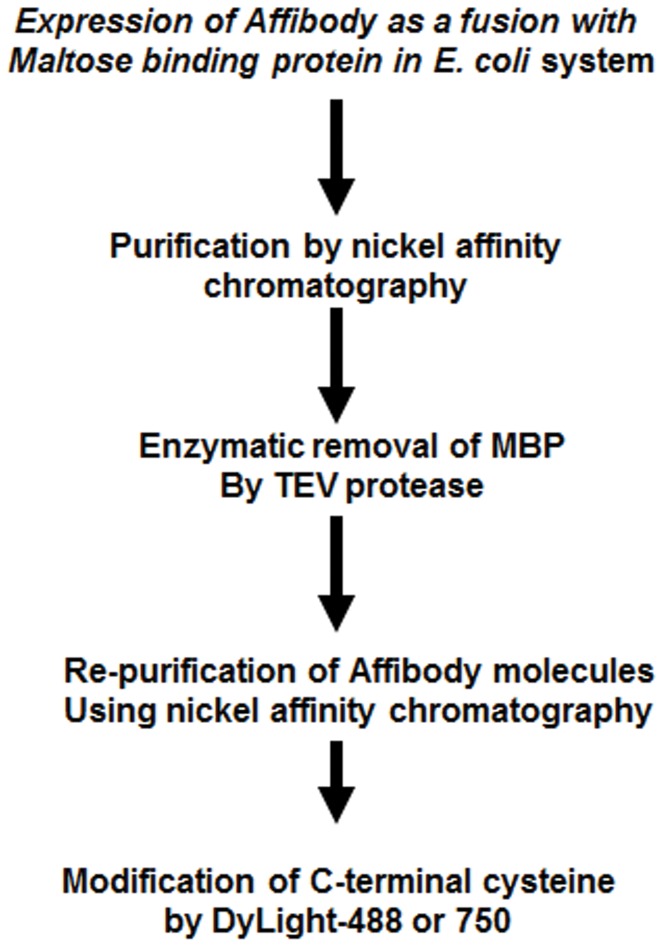
Flow chart of Affibody production and modification.

Both Z_HER2_ and Z_Taq_ (used as a negative control) molecules are based on the same protein Z scaffold and differ only by 12 amino acids (Figure S1). Both constructs were produced as recombinant proteins in an *E. coli* expression system according to the diagram presented in Figure 1. The resulting proteins showed a high level of purity (Figure S2) and the total protein production yield was approximately 4 mg of the protein per one liter of bacterial culture for both molecules. Affibody molecules, containing unique C-terminal cysteine, tend to form dimers in oxidizing environments, and the introduction of a reducing agent (Invitrogen, Carlsbad) during the denaturation step led to monomerization as shown by SDS PAGE (Figure S2). Obtained Affibody molecules were successfully labeled with a fluorescent beacon. Estimated labeling efficiency was 0.6 and 0.4 for DyLight-488 and DyLight-750 respectively. The resulting tracers were characterized *in vitro* (DyLight-488) and *in vivo* (DyLight-750) studies.

### Binding Affinity and Specificity

**Figure 2:**
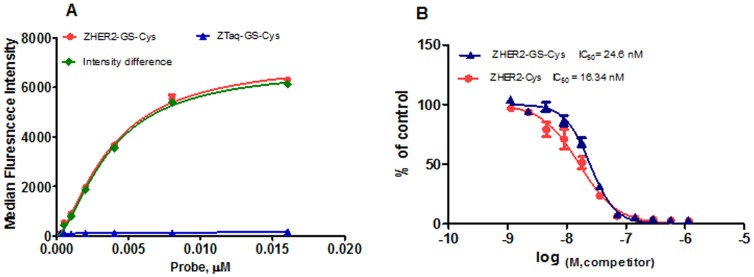
Z_HER_2 binding affinity. A. BT-474 cells were incubated for one hour with increasing concentration of Z_HER2_-GSC-DyLight-488 or Z_Taq_-GSC-DyLight-488. B. BT-474 cells were pre-incubated with increasing concentration of non-modified Z_HER2_ molecules with or without flexible spacer Z(HER2-GS-Cys and Z_HER2_-Cys respectively), followed by addition of 0.5 µg/ml of Z_HER2_-DyLight-48. Fluorescence intensity was measured in triplicates using LSR II flow cytometer. FlowJo (Tree Star Inc, Ashland, OR) was used for FACS data analysis. K_D_ and IC50 values were calculated using GraphPad Prism (GraphPad Software, Inc., San Diego, CA).

Affinity of DyLight-labeled Z_HER2_ to the receptor was estimated by a cell-based saturation assay using BT-474 breast carcinoma cells. Obtained results showed low-nanomolar affinity to the receptor with a binding constant 3.66±0.26 nM. In contrast, DyLight-labeled Z_Taq_, used as a negative control, did not show any fluorescence signal at the tested concentration range (Figure 2A). Competition studies revealed that modification of Affibody molecules with fluorophore slightly reduced their affinity to receptors. Non-labeled Z_HER2_-GS-Cys half-saturated HER2 at 24±1.19 nM in a presence of labeled Z_HER2_-GS-Cys at concentration 51 nM. The affinity of Z_HER2_-GS-Cys was compared to corresponding molecule without glycine-serine linker (Z_HER2_-Cys) obtained from Affibody, AB (Stockholm, Sweden). The corresponding IC_50_ value for that probe was 16.34±0.97**nM (Figure. 2B).

**Figure 3:**
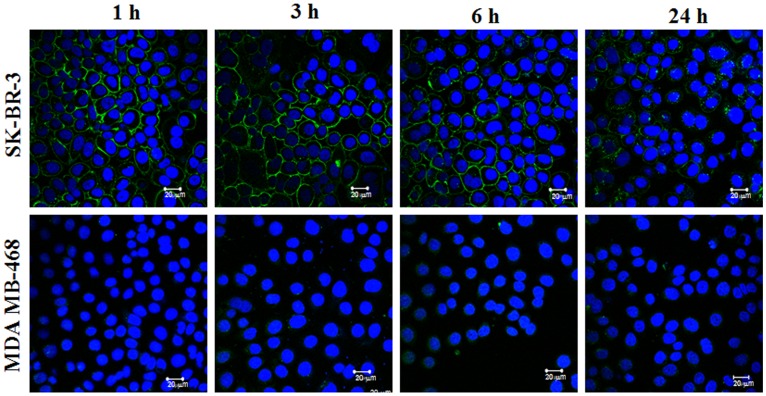
Confocal microscopy of HER2-positive (upper panel) and HER2-negative (lower panel) cells exposed to ZHER2-DyLight-488. SK-BR-3 and MDA-MB-468 cells were plated at 2×10^4^ cells/chamber and exposed to Z_HER2_-DyLight-488 at 0.5 µg/ml. After a 30 min loading step, cells were rinsed and incubation was continued for indicated period of time at 37°C. A half hour before imaging, the nuclei were counterstained with Hoechst 33342 at 0.2 µg/ml. Images were acquired using Zeiss LSM 510 microscope.

Binding specificity was also confirmed by confocal microscopy. Breast cancer cells expressing a high level of HER2 receptors showed evident membrane accumulation of fluorescence after exposure to Z_HER2_-DyLight-488 conjugate. During the course of the experiment no significant internalization of the probe was observed for up to 6 hours of exposure. Interestingly, a membrane-associated signal was still detectable 24 hours after removal of the probe from the incubation medium (Figure 3, upper panel). Neither membrane retention nor intracellular uptake of the probe was observed for HER2-negative MDA-MB-468 cells (Figure 3, lower panel).

As shown in Figure S3, Affibody molecules, either alone or conjugated with DyLight-750, did not affect downstream elements of the EGFR signaling pathway since the phosphorylation level of neither MAPK nor AKT was found upregulated following a 30-min exposure.

### In vivo NIR Optical Imaging

#### Affibody clearance

Affibody-DyLight750 conjugate clearance rate was estimated by scanning the contralateral area of the animal and showed that fluorescence intensity decreased exponentially following Z_HER2_-DyLight-750 injection with an estimated half-life of 37.5±2.8 min. A slightly lower (but not statistically significant) clearance rate was reported for Z_Taq_ -DyLight-750 (43.2±8.2 min, Table S1).

#### Accumulation of the probe in the tumor

**Figure 4:**
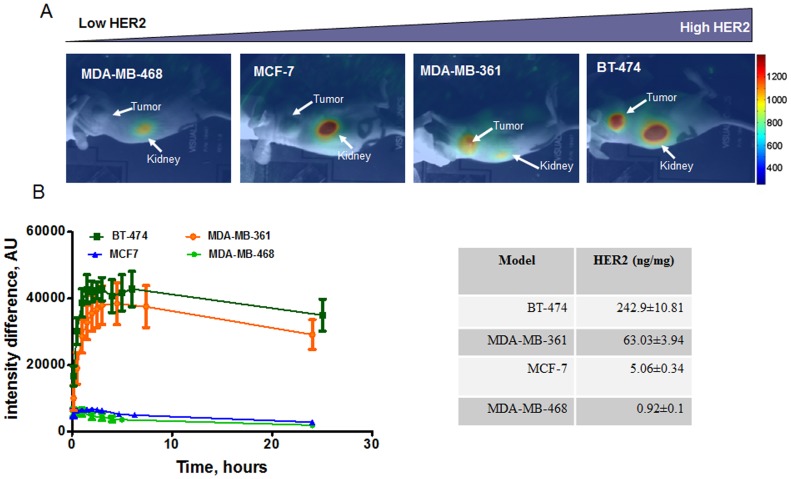
Accumulation of Z_HER2_ in xenografts with different HER2 expression level. 10 µg of Z_HER2_-DyLight-750 conjugate were injected by IV into athymic mice bearing tumors expressing different levels of HER2 (A) CCD camera image of mice three hours post probe administration. (B) Tumor as well as contra-lateral area of animals were scanned at several time points (10 min to 24 h post injection) using in-house system with the scanning head, incorporating a linear array of source and detector fibers. Fluorescence signal is expressed as an intensity difference between tumor and contra-lateral site as described in Material and Methods Section. Table insert presents the level of HER2 measured in extracted tissue using HER2 ELISA and is expressed as nanogram of HER2 per mg of total protein.

Accumulation of the probe in the tumor was observed for HER2-positive tumors only. The highest fluorescence intensity was reported for the BT-474 model, followed by MDA-MB-361. Low or no accumulation was observed for MCF-7 and the HER2-negative MDA-MB-468 model (Figure 4A and B). The level of HER2 expression was measured *ex vivo* in extracted tumor tissue and is presented as an insert on Figure 4.

Interestingly, the maximal accumulation in BT-474 tumors was observed approximately 90 minutes post probe administration, while more than 3 hours were required to reach the peak signal in MDA-MB-361 xenografts. In both cases, signals decreased slowly to ∼80% of the peak value at 24 hours post injection (Figure 4B).

**Figure 5:**
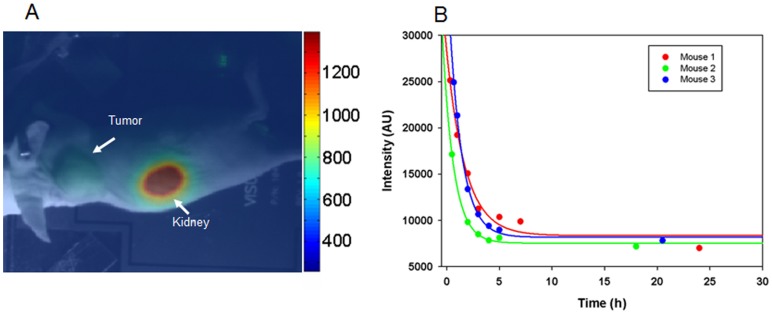
Distribution and kinetics of fluorescence signal in mice with HER2-positive tumor injected with "off-target" Affibody-DyLight-750 conjugate. A. Representative CCD camera image of BT-474 mouse three hours post injection with 10 µg of Z_Taq_-Affibody-DyLight-750 conjugate. B. One-phase decay function 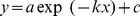 in MatLab software was used to quantify fluorescence kinetic. Blue, red, and green represent signal quantified for each mouse in the experiment.

No probe accumulation was observed when mice bearing HER2-positive tumors (BT-474) were injected with “off-target” Affibody molecules (Z_Taq_) labeled with DyLight-750 (Figure 5B).

In order to further illustrate the real-time accumulation of Affibody in HER2-positive tumor, we used Pearl Impulse Imaging System. Mice, bearing BT-474 tumors, were catheterized and positioned in the imager before injection. Probe injection was done during image acquisition. As presented in Movie S1, fluorescent probe was immediately distributed throughout the body following administration. Evident kidney accumulation of the signal was observed as early as one minute post injection, followed by fluorescence accumulation in the tumor volume. Interestingly, images taken 24, 48, 72 hours, and five days post injection revealed that tumor-originated fluorescence could be detected even a few days post probe injection (Figure S4).

As shown in Figures 4 and 5, and Figure S5, animals receiving fluorescently labeled Affibody molecules (both HER2-specific and non-specific) showed evident signal accumulation in the kidney area. Interestingly, fluorescence accumulation in the kidneys had a semi-permanent character and could be detected even few days post probe administration as shown in long term imaging experiments (Figure S4).

**Figure 6:**
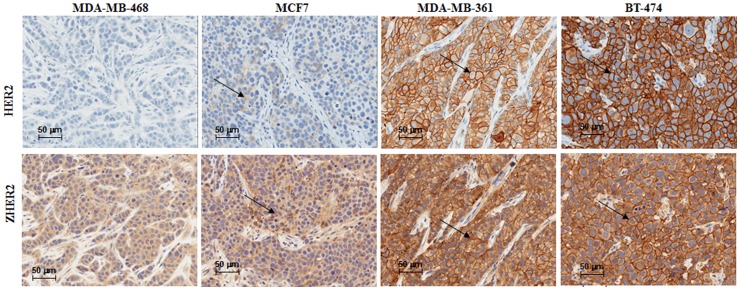
IHC analysis of tumor tissue extracted from four different breast tumor models 24 hours post Z_HER2_-DyLight-750 injection. Tumor tissues were extracted from animals 24 hours post probe injection, fixed in 10%NBF and analyzed by IHC. Adjacent sections were stained for detection of HER2 (upper panel) and Affibody as described in Material and Methods section. Arrows point positively stained cell membranes in the tumor tissue.

HER2-dependent Affibody accumulation was confirmed by IHC analysis. Specimens extracted from mice bearing BT-474 and MDA-MB-361 tumors showed positive membrane staining for anti-HER2 antibodies, while MCF7 and MDA-MB-468 showed very weak or no membrane signal, respectively (Figure 6, upper panel). Similar staining pattern was obtained using anti-Affibody antibodies for tumors extracted 24 hours post-probe injection (Figure 6, lower panel).

### Characterization of HER2 Receptor Expression in vivo

**Figure 7:**
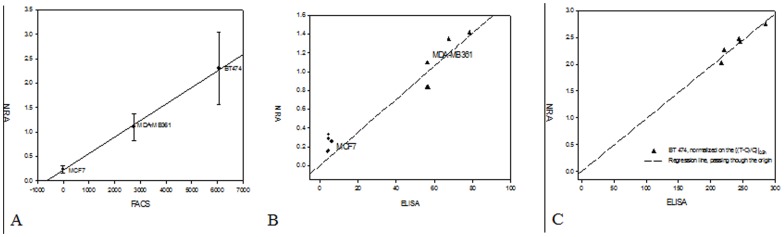
Relationship between normalized rates of accumulation of the probe and HER2 level. A. Relationship between normalized rates of accumulation of the probe and HER2 level (hours) in three types of HER2-positive tumors (BT-474, MDA-MB361, MCF7), estimated from *in vivo* fluorescence imaging and Affibody-DL-488 retention for corresponding cell lines to the same probe, found *in vitro*, using FACS **B.** Relationship between normalized rates of accumulation of the probe in individual HER2-positive tumors (MDA-MB-361, MCF7), estimated from *in vivo* fluorescence imaging and ELISA readings for the same tumor (ng/mg), dashed line is linear regression of MDA-MB-361 data, passing through the origin **C.** Relationship between normalized rates of accumulation of the probe in individual HER2 tumor BT474 (3+), estimated from *in vivo* fluorescence imaging and ELISA readings for the same tumor, dashed line is the linear regression, passing through the origin.

In order to characterize HER2 expression *in vivo* we employed dynamic imaging approach and mathematical analysis as described in [28], [31] and Material and Methods section. For each type of the tumor, average Normalize Rate of Accumulation (NRA) values were calculated and compared with the Affibody-DyLight-488 retention measured by flow cytometry (FACS). For each used cancer cell line the results, presented in Figure 7a, indicate a good linear correlation between both parameters over the broad range of HER2 overexpression in cancer cells.

In Figure 7b, we present comparison between NRA values for lower HER2 expression carcinomas MCF7 and MDA-MB-361 and ELISA readings for the same tumor in the case of Affibody-DyLight750 probe. The first approximation relationship between two parameters can be considered linear. Corresponding p-value for the statistical test of the null hypotheses is equal to 4.67e-08, substantiating non-zero value of the linear regression slope.

In the case of BT-474 (the highest HER2 expression) we should take into account large variations in the angiogenesis between individual tumors, resulting in different amounts of free ligand in blood in the tumor area, as has been described in our recent manuscript [28]. Therefore, we have normalized the difference between tumor and contralateral intensities by its values at early times (*t* = 0.5 h) before fitting them to the kinetic model, describing binding of specific probe to receptors. Resulting relationship between the data obtained by NRA and ELISA proved to be close to linear dependence with zero intercept (Figure 7C). Corresponding p-value for the test of the null hypotheses is equal to 3.9e-07, showing that statistically the found slope of this line is significantly different from zero. Moreover, according to Akaike information criterion (AIC) the statistical model with zero intercept (AIC = 38.7) is better than the linear regression with the intercept, as a fitting parameter (AIC = 40.5) [32].

## Discussion

In order to create a new, improved Affibody-based florescent probe we used a monovalent Z_HER2_ molecule, with the C-terminal cysteine separated by a flexible glycine-serine (GS) linker. We believe that incorporation of that spacer decreased steric hindrance between the receptor and the C-terminal fluorescent beacon. Such a re-reengineered Affibody molecule, after modification with DyLight fluorescent dye, showed high affinity to HER2. Based on *in vitro* saturation experiments, we conclude that modification of the C-terminal part of Z_HER2_ molecules with fluorescent dye resulted in a moderate decrease of affinity (the K_D_ value reduced twice as compared to non-modified Affibodies), but was still approximately three- to four- fold higher than the one reported for Affibody molecules modified with Fluorine-18 for PET imaging [17] or for Z_HER2_ fusions with EGFP or mCherry proteins [19]. On the other hand, the dissociation constant was significantly higher than that reported for monovalent Z_HER2_ obtained in surface plasmon resonance-based experiments [12]. For imaging, it is important that applied tracers do not interfere with the system. It was shown that Z_HER2_ molecules neither induce toxic effects on breast cancer cells [27] or normal lung fibroblast (Figure S6) nor trigger activation of EGFR signaling as the phosphorylation levels of MAPK and AKT were not upregulated after exposure to Affibody (Figure S3). Limited internalization of HER2-Affibody-is consistent with the previous observation, even after relatively long exposures [19], [33].

In contrast to the divalent Z_HER2_-Albumin binding domain hybrid tracer, used in our previous study, the monovalent Affibody was efficiently cleared from the circulation with a half-life of approximately 30–40 minutes observed for both HER2-specific and non-specific probes. A similar rate of Affibody clearance was reported for tracers labeled with Fluorine 18 [17] and Gallium-68 [34]. The rapid Affibody molecule clearence is most likely due to glomerular filtration and is consistent with the relatively low molecular size of the tracer. Interestingly, unlike for F-18 or Ga-68 -Labeled Affibody signal accumulation in the kidneys was persistent (Figure S4). Since Affibody molecules used in this study do not cross-react with murine receptor and non-HER2-specific Affibodies display similar kidneys accumulation pattern (not shown), we conclude that observed phenomenon results from the nature of the fluorescent beacon rather than HER2-driven interaction. Delineation of the molecular foundation for that observation and its potential long-term toxicity implications requires, however, further studies.

*In vivo* evaluation of Z_HER2_-DyLight-750 binding to breast cancer xenografts confirmed our *in vitro* observations. The probe accumulation in the tumors was HER2-dependent, since its non-HER2-specific analog failed to show any retention. In a similar vein HER2-negative xenografts (MDA-MB-468) did not retain the fluorescent signal in the tumor area. Surprisingly, the relation between maximum fluorescence uptake (maximum concentration of the bound fluorescent ligands in region of interest (ROI) over the tumor) and HER2 expression level was not very well correlated when the total HER2 level determined by ELISA was taken into consideration (R^2^ = 0.67) (Figure S5). This might be due to the fact that, besides the level of recognizable receptor on the tumor cells, other factors also affect probe delivery. These may include, but are not limited to, the density of the tumor vasculature and the hydrostatic pressure of interstitial fluid [35]. To better describe the relation between receptor expression and probe accumulation patterns we have developed a mathematical kinetic model [28], [31]. Using this model, we have shown that fluorescence imaging, using HER2-specific Affibody-DyLight conjugates as a contrast agent, allow assessment of HER2 expression by analysis of time series of fluorescent images. Relationship between average NRA and FACS measurements for three types of breast carcinoma is close to linear, indicating that our method may be used to evaluate the receptor expression level in different types of tumor. This capacity might be applied to assess HER2 expression during the course of HER2-targeted therapy for treatment monitoring and optimization, especially in the pre-clinical stage of drug development.

Overall, our data indicate that optical imaging using Z_HER2_ molecules labeled with a NIR beacon may provide robust information about HER2 status in pre-clinical models in a reliable and cost-effective manner, avoiding exposure to ionizing radiation. In the future, it might become a convenient, complementary tool for selection of breast cancer patients for HER2-targeted therapies if validated in clinical trials. Due to its minimally-invasive character, this method might be also used for sequential monitoring of the response of individual tumors to therapies. Finally, Affibody molecules, conjugated with appropriate fluorescent tracers, could be potentially applied to increase the contrast between normal and HER2-overexpressing malignant tissue and, thereby, facilitate detection of HER2-positive metastatic lesions during NIR-assisted surgery.

## Supporting Information

Figure S1 **Sequence of HER2- and Taq-specific Affibody molecules fused to MBP.** Maltose binding protein sequence is marked by blue font, sequence recognized by TEV-protease-green. Red font represents aminoacids unique for Taq-specific Affibody Molecules. Brown font marks flexible glycine-serine linker separating unique C-terminal cysteine.
(TIF)

Figure S2 **Electrophoretic analysis of purified Z_HER2_ (A) and Z_Taq_ (B).** 10 µg of protein were resolved in 4–12% PAGE. Samples were reduced before separation (lane 1) or resolved without reduction (lane 2).
(TIF)

Figure S3 **Western blot analysis of MAPK and AKT after exposure to Affiobody molecules.** Starved SKBR3 cells were exposed for 30 min to Affibody molecules and their fluorescent derivative. For positive control cells were treated with EGF. 20 µg of cell lysates was separated in 4–12% NU PAGE followed by transfer on PVDF membrane. Immunodetection was performed using anti-phosphorylated MAPK (D13.14.4E) and AKT (244F9) antibodies. Anti-total MAPK (137F5) and anti-total AKT (11E7) were used for detection of the protein is cell lysates. All Antibodies were from Cell Signaling Technology.
(TIF)

Figure S4 **Fluorescent images acquired at different time intervals post Z_HER2_-DyLight-750 probe injection.** CCD camera image of mouse bearing BT-474 tumors at different time post injection of 10 µg of HER2-Affibody-DyLight-750 conjugate. Images were acquired using Pearl Impulse Imaging System (LI-COR Biosciences).
(TIF)

Figure S5 **Correlation between receptor expression and maximum HER2-AffibodyDyLight-750 uptake.** Receptor expression in MDA-MB-468, MCF-7, MDA-MB-361 and BT-474 was performed on extracted tumor tissue by ELISA (expressed as nanogram of HER2 per milligram of the tissue lysate) and plotted against maximum uptake of HER2-AffibodyDyLight-750 recorded for different xenograft.
(TIF)

Figure S6 **Toxicity of Z_HER2_ and Z_Taq_ Affibodies tested on lung fibroblast.** WI38 fibroblast were plated on 96-well plate at 5×10^2^ cells/well. After overnight attachment, cells were treated with indicated concentrations of Z_HER2_ and Z_Taq_ Affibodies for 72 hours. Viability was measured using CellTiter-Glo, Promega, Madison, WI). Treated cells were normalized to vehicle-treated control.
(TIF)

Table S1 **Half-life of the probe determined based on changes of fluorescence signal measured on contralateral site as a function of time.** Mice were injected IV with 10 µg of ZHER2-DyLight-750 conjugate (A) or Taq-Affibody-DyLight-750 (B). Fluorescence intensity over ROI on contralateral site was measured as described before. Half-life was calculated using one-phase decay function in GraphPad Prism software.
(TIF)

Movie S1 **Continuous monitoring of fluorescence distribution in mouse bearing s.c. BT-474 tumor injected with Z_HER2_-DyLight-750 conjugate.** Mouse were positioned in Pearl Impulse Imaging System (LI-COR Biosciences), catheterized, and injected with 10 µg of Affibody-DyLight-750 conjugate. Series images were taken every second for 1 min, followed by 2-hours imaging with two frames per minute. During imaging mouse was kept at shallow anesthesia (1%, isoflurane) and sacrificed after the imaging was accomplished.
(WMV)

## References

[1] Menard S, Pupa SM, Campiglio M, Tagliabue E (2003). Biologic and therapeutic role of HER2 in cancer.. Oncogene.

[2] Meric-Bernstam F, Hung MC (2006). Advances in targeting human epidermal growth factor receptor-2 signaling for cancer therapy.. Clin Cancer Res.

[3] Witton CJ, Reeves JR, Going JJ, Cooke TG, Bartlett JM (2003). Expression of the HER1–4 family of receptor tyrosine kinases in breast cancer.. J Pathol.

[4] Slamon DJ, Clark GM, Wong SG, Levin WJ, Ullrich A (1987). Human breast cancer: correlation of relapse and survival with amplification of the HER-2/neu oncogene.. Science.

[5] Schuetz F (2011). Adjuvant Systemic Therapy of Breast Cancer.. Breast Care (Basel).

[6] Allison M (2010). The HER2 testing conundrum.. Nat Biotechnol.

[7] Nord K, Gunneriusson E, Ringdahl J, Stahl S, Uhlen M (1997). Binding proteins selected from combinatorial libraries of an alpha-helical bacterial receptor domain.. Nat Biotechnol.

[8] Nygren PA (2008). Alternative binding proteins: affibody binding proteins developed from a small three-helix bundle scaffold.. FEBS J.

[9] Urica N (2008). EGFR and HER2 Targeting for Radionuclide-Based Imaging and Therapy: Preclinical Studies.. Uppsala University, Medicinska vetenskapsområdet, Faculty of Medicine, Department of Oncology, Radiology and Clinical Immunology, Biomedical PhD.

[10] Eigenbrot C, Ultsch M, Dubnovitsky A, Abrahmsen L, Hard T (2010). Structural basis for high-affinity HER2 receptor binding by an engineered protein.. Proc Natl Acad Sci U S A.

[11] Orlova A, Tolmachev V, Pehrson R, Lindborg M, Tran T (2007). Synthetic affibody molecules: a novel class of affinity ligands for molecular imaging of HER2-expressing malignant tumors.. Cancer Res.

[12] Ekblad T, Tran T, Orlova A, Widstrom C, Feldwisch J (2008). Development and preclinical characterisation of 99mTc-labelled Affibody molecules with reduced renal uptake.. Eur J Nucl Med Mol Imaging.

[13] Tran T, Engfeldt T, Orlova A, Sandstrom M, Feldwisch J (2007). (99 m)Tc-maEEE-Z(HER2:342), an Affibody molecule-based tracer for the detection of HER2 expression in malignant tumors.. Bioconjug Chem.

[14] Tolmachev V, Nilsson FY, Widstrom C, Andersson K, Rosik D (2006). 111In-benzyl-DTPA-ZHER2:342, an affibody-based conjugate for in vivo imaging of HER2 expression in malignant tumors.. J Nucl Med.

[15] Tolmachev V, Friedman M, Sandstrom M, Eriksson TL, Rosik D (2009). Affibody molecules for epidermal growth factor receptor targeting in vivo: aspects of dimerization and labeling chemistry.. J Nucl Med.

[16] Nordberg E, Orlova A, Friedman M, Tolmachev V, Stahl S (2008). In vivo and in vitro uptake of 111In, delivered with the affibody molecule (ZEGFR:955)2, in EGFR expressing tumour cells.. Oncol Rep.

[17] Kramer-Marek G, Kiesewetter DO, Martiniova L, Jagoda E, Lee SB (2008). [18F]FBEM-Z(HER2:342)-Affibody molecule-a new molecular tracer for in vivo monitoring of HER2 expression by positron emission tomography.. Eur J Nucl Med Mol Imaging.

[18] Lundberg E, Hoiden-Guthenberg I, Larsson B, Uhlen M, Graslund T (2007). Site-specifically conjugated anti-HER2 Affibody molecules as one-step reagents for target expression analyses on cells and xenograft samples.. J Immunol Methods.

[19] Lyakhov I, Zielinski R, Kuban M, Kramer-Marek G, Fisher R (2010). HER2- and EGFR-specific affiprobes: novel recombinant optical probes for cell imaging.. Chembiochem.

[20] Puri A, Kramer-Marek G, Campbell-Massa R, Yavlovich A, Tele SC (2008). HER2-specific affibody-conjugated thermosensitive liposomes (Affisomes) for improved delivery of anticancer agents.. J Liposome Res.

[21] Smith B, Lyakhov I, Loomis K, Needle D, Baxa U, et al. Hyperthermia-triggered intracellular delivery of anticancer agent to HER2(+) cells by HER2-specific affibody (ZHER2-GS-Cys)-conjugated thermosensitive liposomes (HER2(+) affisomes).. J Control Release.

[22] Zielinski R, Lyakhov I, Jacobs A, Chertov O, Kramer-Marek G (2009). Affitoxin–a novel recombinant, HER2-specific, anticancer agent for targeted therapy of HER2-positive tumors.. J Immunother.

[23] Zielinski R, Lyakhov I, Hassan M, Kuban M, Shafer-Weaver K (2011). HER2-affitoxin: a potent therapeutic agent for the treatment of HER2-overexpressing tumors.. Clin Cancer Res.

[24] Hoffman RM (2005). The multiple uses of fluorescent proteins to visualize cancer in vivo.. Nat Rev Cancer.

[25] Shaner NC, Steinbach PA, Tsien RY (2005). A guide to choosing fluorescent proteins.. Nat Methods.

[26] Ardeshirpour Y, Chernomordik V, Capala J, Hassan M, Zielinsky R (2011). Using in-vivo fluorescence imaging in personalized cancer diagnostics and therapy, an image and treat paradigm.. Technol Cancer Res Treat.

[27] Lee SB, Hassan M, Fisher R, Chertov O, Chernomordik V (2008). Affibody molecules for in vivo characterization of HER2-positive tumors by near-infrared imaging.. Clin Cancer Res.

[28] Hassan M, Chernomordik V, Zielinski R, Ardeshirpour Y, Capala J (2012). In Vivo Method to Monitor Changes in Her2 Expression Using Near-infrared Fluorescence Imaging.. Mol Imaging.

[29] Kapust RB, Tozser J, Fox JD, Anderson DE, Cherry S (2001). Tobacco etch virus protease: mechanism of autolysis and rational design of stable mutants with wild-type catalytic proficiency.. Protein Eng.

[30] Hassan M, Riley J, Chernomordik V, Smith P, Pursley R (2007). Fluorescence lifetime imaging system for in vivo studies.. Mol Imaging.

[31] Chernomordik V, Hassan M, Lee SB, Zielinski R, Gandjbakhche A (2010). Quantitative analysis of Her2 receptor expression in vivo by near-infrared optical imaging.. Mol Imaging.

[32] Crawley MJ (2005). Statistics: An Introduction Using R. London: John Wiley & Sons, Ltd..

[33] Tran TA, Rosik D, Abrahmsen L, Sandstrom M, Sjoberg A (2009). Design, synthesis and biological evaluation of a multifunctional HER2-specific Affibody molecule for molecular imaging.. Eur J Nucl Med Mol Imaging.

[34] Kramer-Marek G, Shenoy N, Seidel J, Griffiths GL, Choyke P, et al. (68)Ga-DOTA-Affibody molecule for in vivo assessment of HER2/neu expression with PET.. Eur J Nucl Med Mol Imaging.

[35] Hofmann M, Guschel M, Bernd A, Bereiter-Hahn J, Kaufmann R (2006). Lowering of tumor interstitial fluid pressure reduces tumor cell proliferation in a xenograft tumor model.. Neoplasia.

