# Supplementary figures and images for: Affibody-DyLight Conjugates for In Vivo Assessment of HER2 Expression by Near-Infrared Optical Imaging
Source: PLoS One. 2012 Jul 20;7(7):e41016. doi: 10.1371/journal.pone.0041016 (PMC3401287; doi:10.1371/journal.pone.0041016)

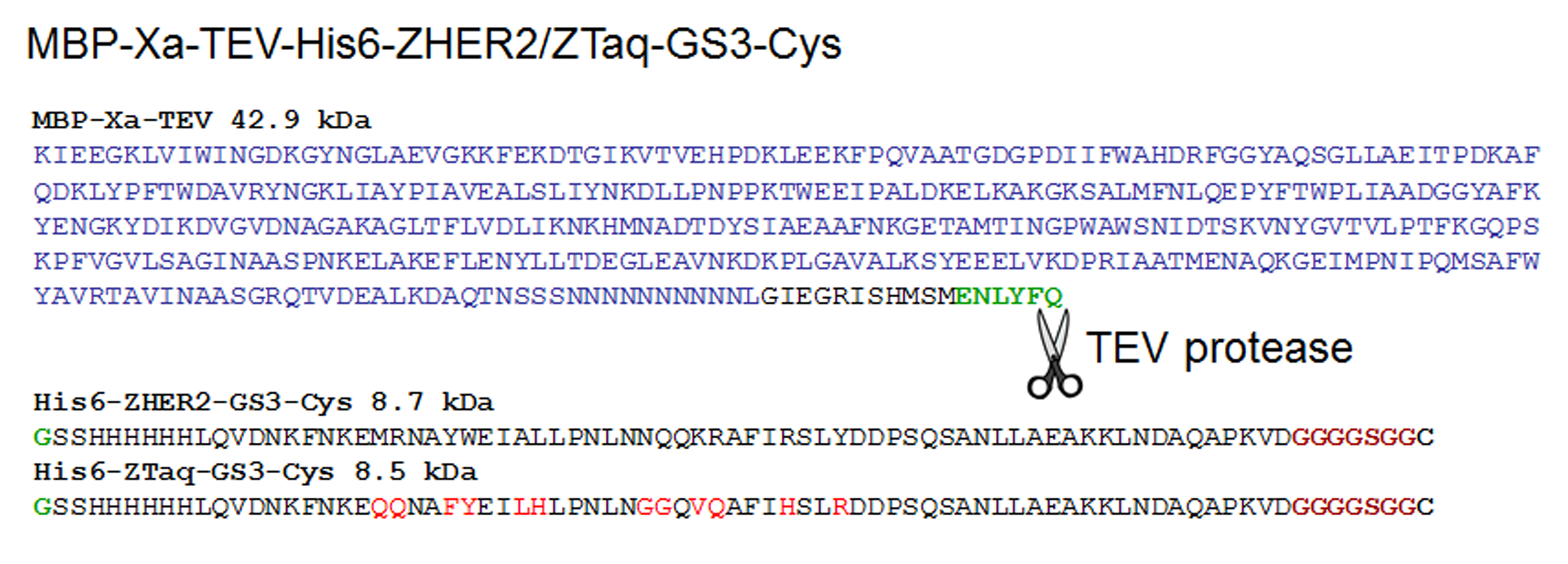

Supplement: Figure S1 — Sequence of HER2- and Taq-specific Affibody molecules fused to MBP. Maltose binding protein sequence is marked by blue font, sequence recognized by TEV-protease-green. Red font represents aminoacids unique for Taq-specific Affibody Molecules. Brown font marks flexible glycine-serine linker separating unique C-terminal cysteine. (TIF) [file pone.0041016.s001.tif]

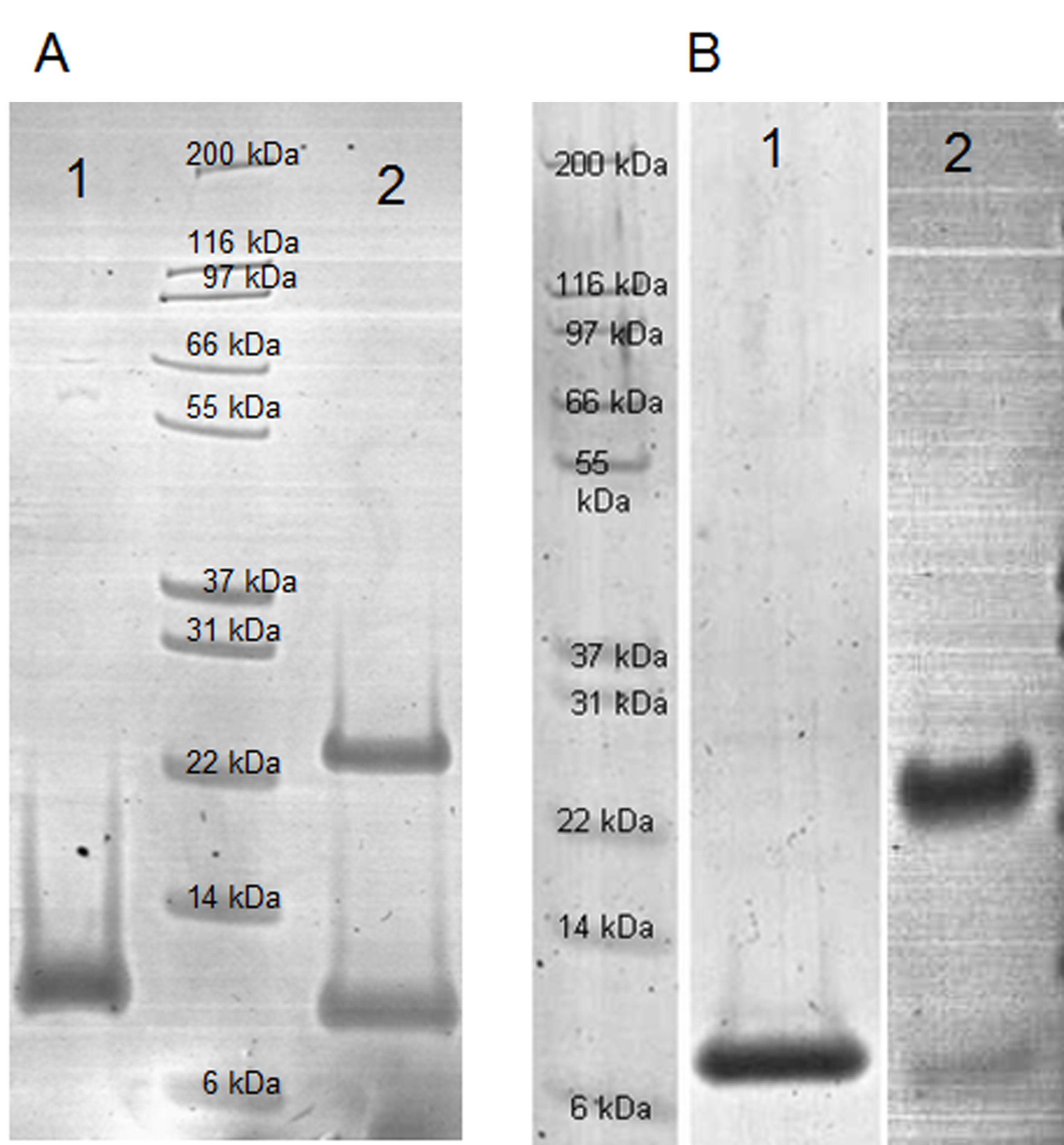

Supplement: Figure S2 — Electrophoretic analysis of purified ZHER2 (A) and ZTaq (B). 10 µg of protein were resolved in 4–12% PAGE. Samples were reduced before separation (lane 1) or resolved without reduction (lane 2). (TIF) [file pone.0041016.s002.tif]

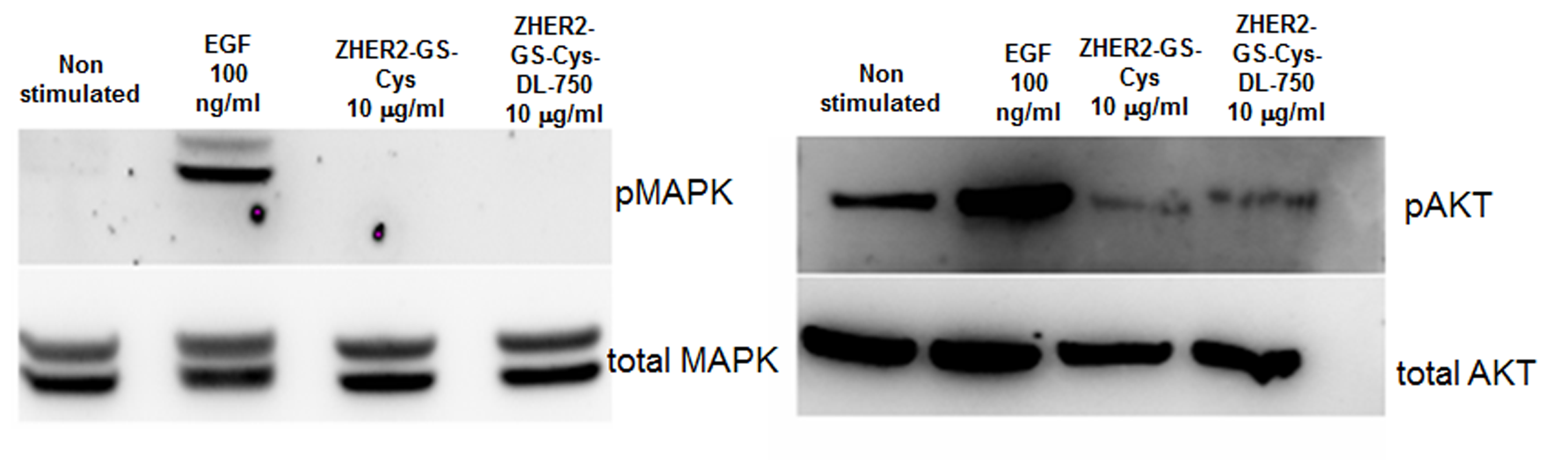

Supplement: Figure S3 — Western blot analysis of MAPK and AKT after exposure to Affiobody molecules. Starved SKBR3 cells were exposed for 30 min to Affibody molecules and their fluorescent derivative. For positive control cells were treated with EGF. 20 µg of cell lysates was separated in 4–12% NU PAGE followed by transfer on PVDF membrane. Immunodetection was performed using anti-phosphorylated MAPK (D13.14.4E) and AKT (244F9) antibodies. Anti-total MAPK (137F5) and anti-total AKT (11E7) were used for detection of the protein is cell lysates. All Antibodies were from Cell Signaling Technology. (TIF) [file pone.0041016.s003.tif]

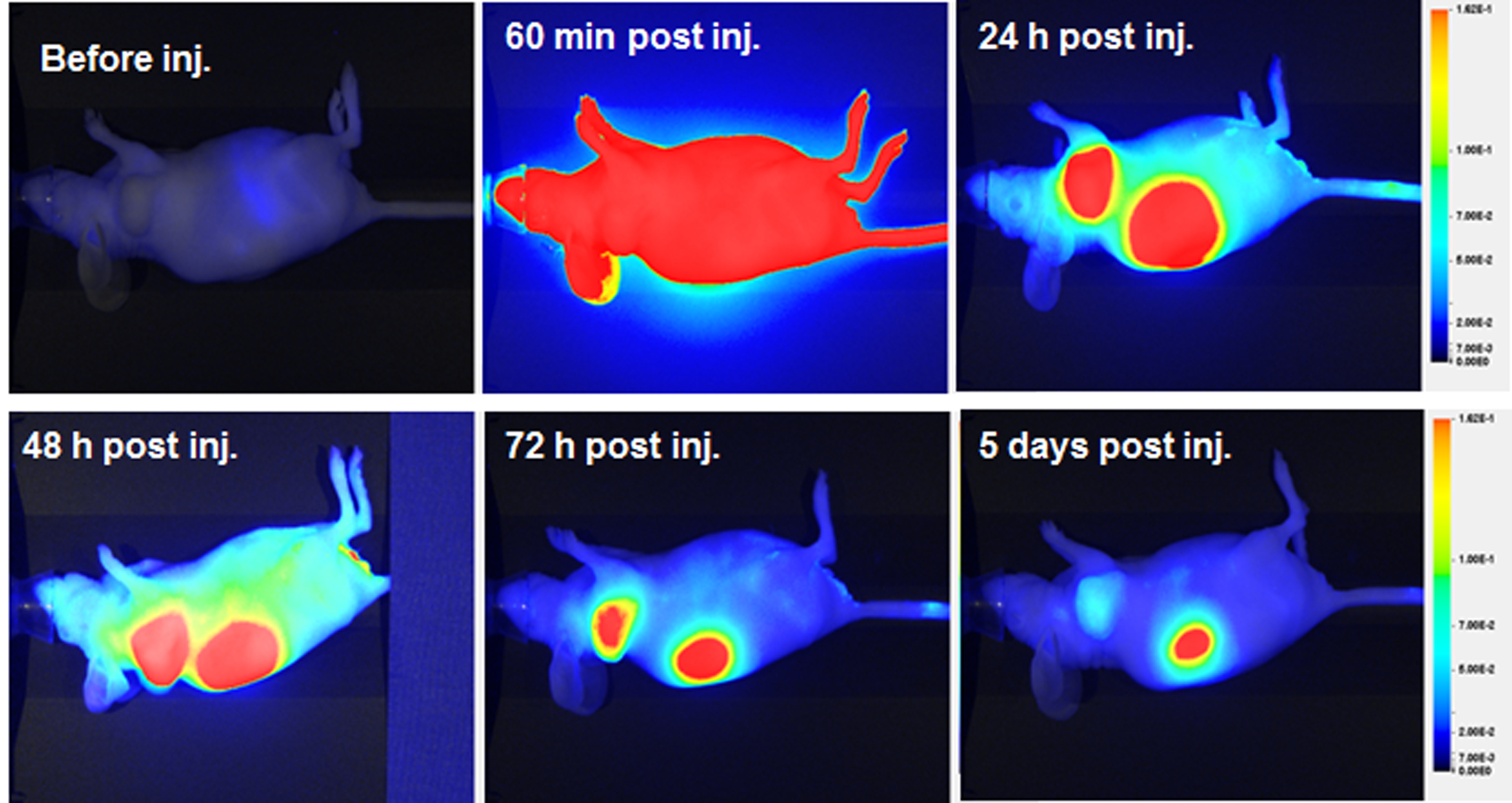

Supplement: Figure S4 — Fluorescent images acquired at different time intervals post ZHER2-DyLight-750 probe injection. CCD camera image of mouse bearing BT-474 tumors at different time post injection of 10 µg of HER2-Affibody-DyLight-750 conjugate. Images were acquired using Pearl Impulse Imaging System (LI-COR Biosciences). (TIF) [file pone.0041016.s004.tif]

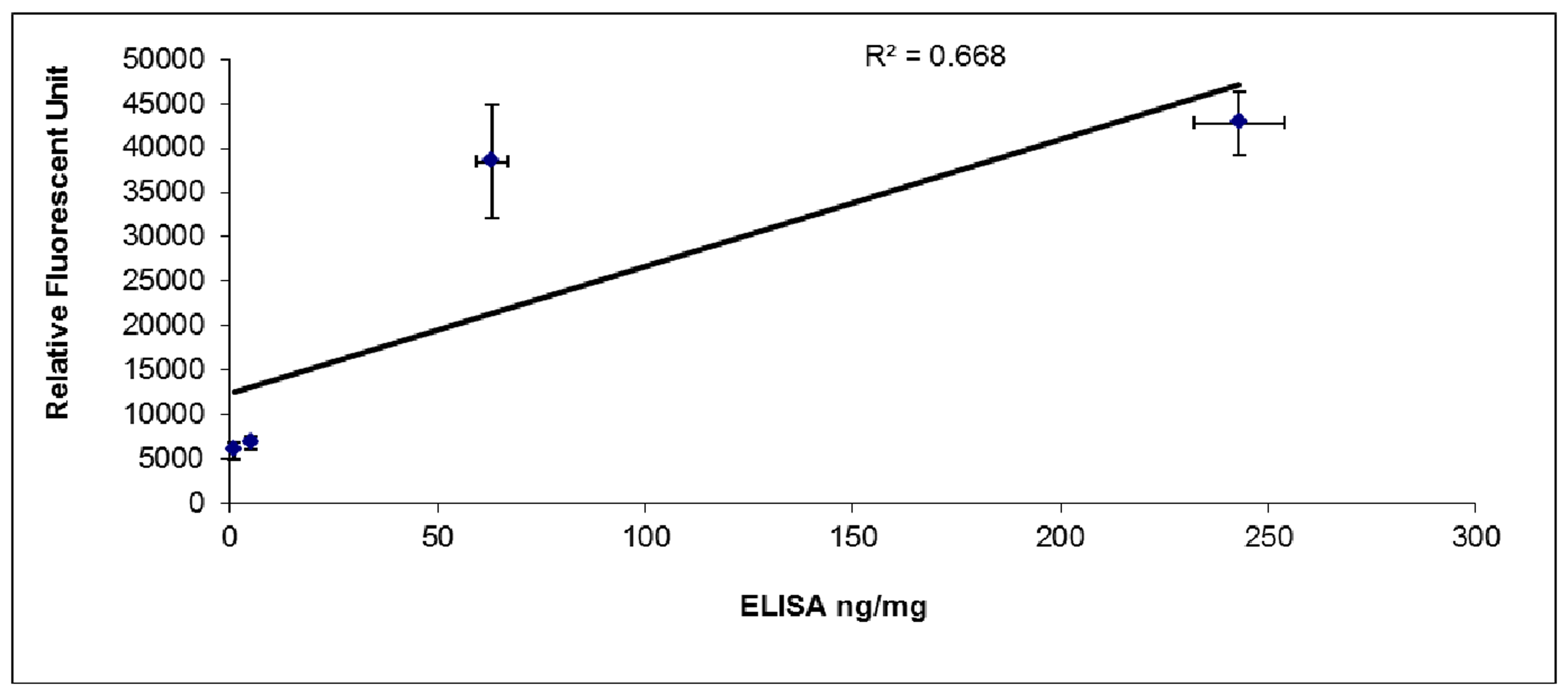

Supplement: Figure S5 — Correlation between receptor expression and maximum HER2-AffibodyDyLight-750 uptake. Receptor expression in MDA-MB-468, MCF-7, MDA-MB-361 and BT-474 was performed on extracted tumor tissue by ELISA (expressed as nanogram of HER2 per milligram of the tissue lysate) and plotted against maximum uptake of HER2-AffibodyDyLight-750 recorded for different xenograft. (TIF) [file pone.0041016.s005.tif]

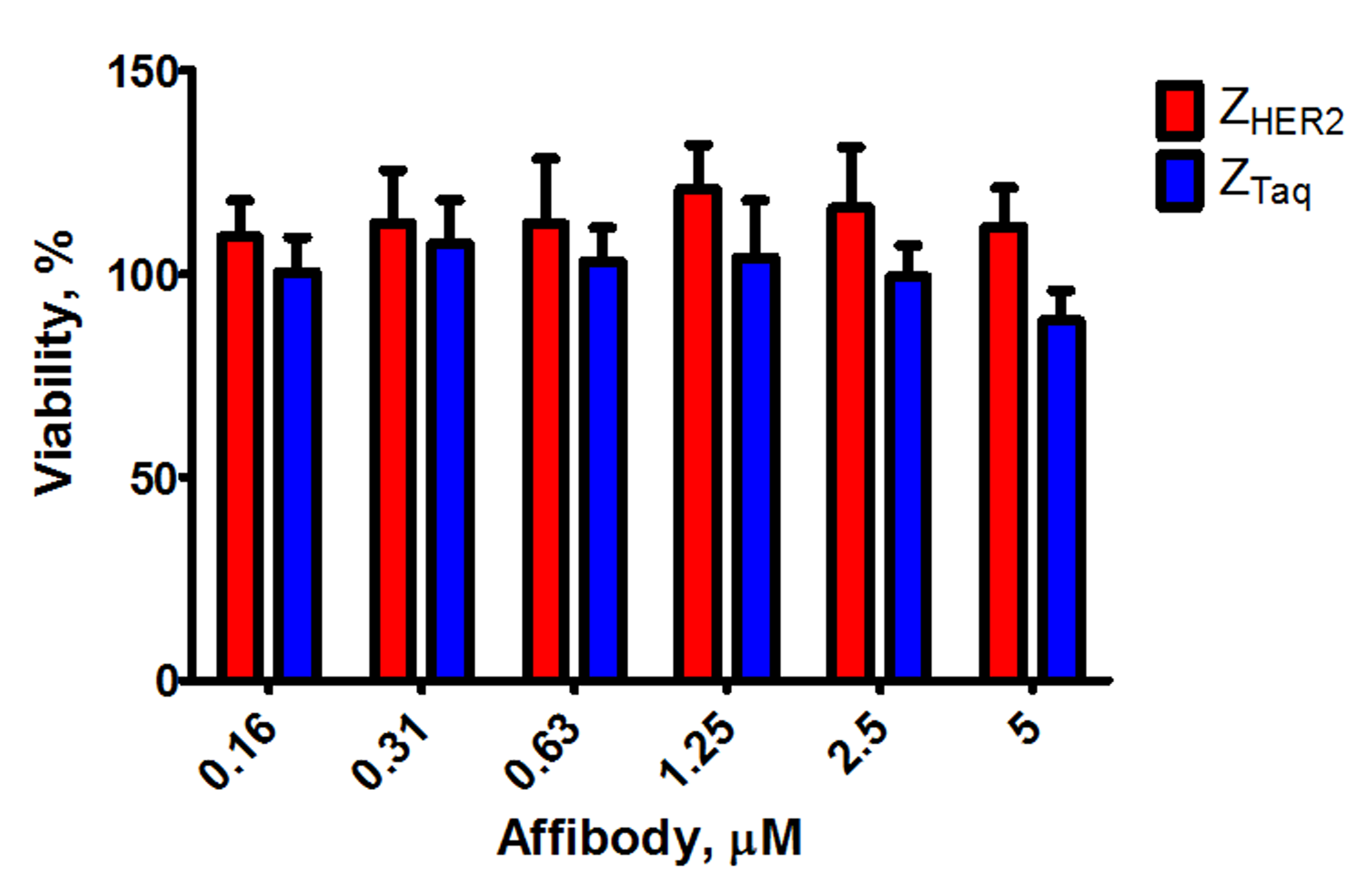

Supplement: Figure S6 — Toxicity of ZHER2 and ZTaq Affibodies tested on lung fibroblast. WI38 fibroblast were plated on 96-well plate at 5×10^2^ cells/well. After overnight attachment, cells were treated with indicated concentrations of ZHER2 and ZTaq Affibodies for 72 hours. Viability was measured using CellTiter-Glo, Promega, Madison, WI). Treated cells were normalized to vehicle-treated control. (TIF) [file pone.0041016.s006.tif]

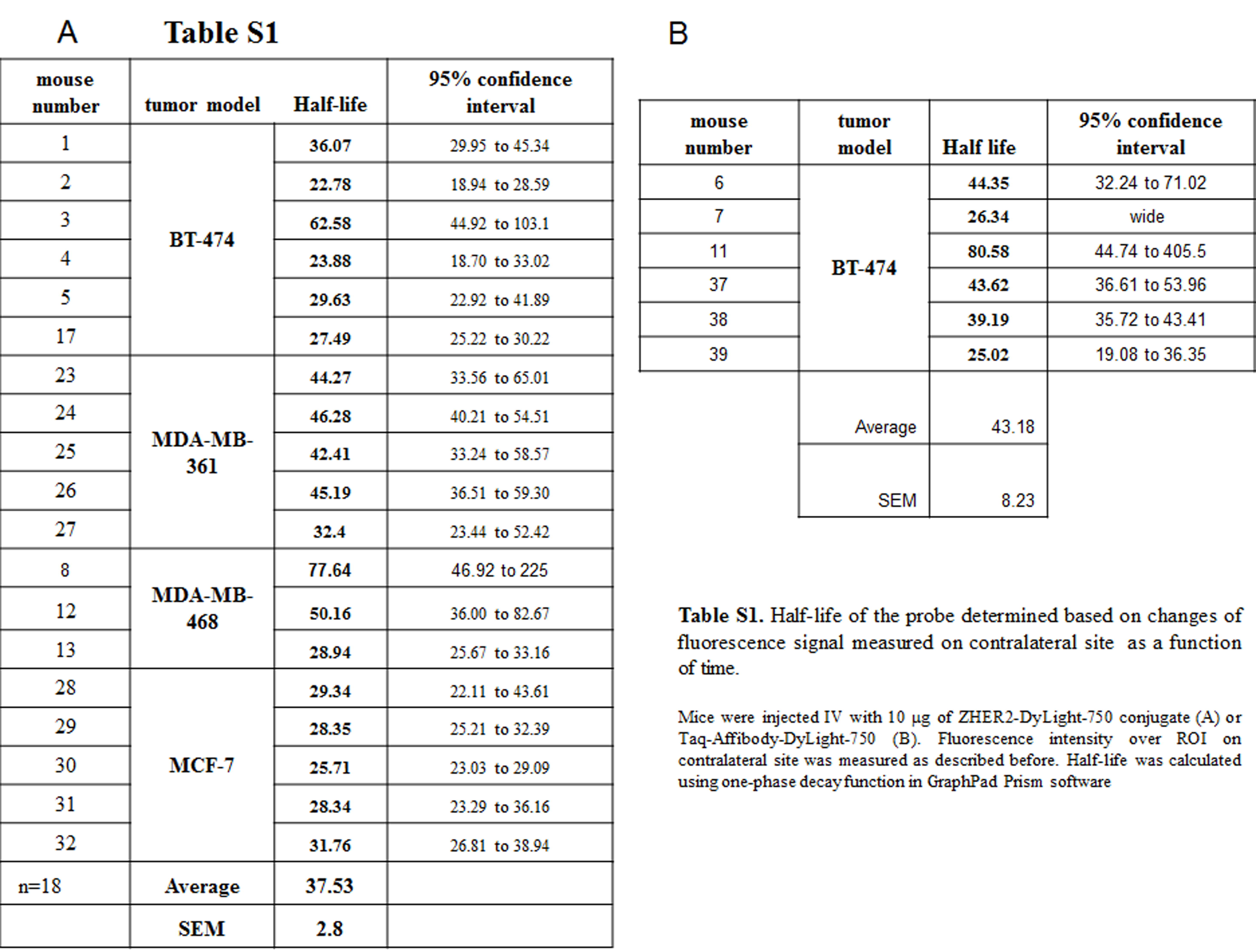

Supplement: Table S1 — Half-life of the probe determined based on changes of fluorescence signal measured on contralateral site as a function of time. Mice were injected IV with 10 µg of ZHER2-DyLight-750 conjugate (A) or Taq-Affibody-DyLight-750 (B). Fluorescence intensity over ROI on contralateral site was measured as described before. Half-life was calculated using one-phase decay function in GraphPad Prism software. (TIF) [file pone.0041016.s007.tif]
